# Visualizing Rous Sarcoma Virus Genomic RNA Dimerization in the Nucleus, Cytoplasm, and at the Plasma Membrane

**DOI:** 10.3390/v13050903

**Published:** 2021-05-13

**Authors:** Eunice C. Chen, Rebecca J. Kaddis Maldonado, Leslie J. Parent

**Affiliations:** 1 Department of Medicine, Division of Infectious Diseases and Epidemiology, Penn State College of Medicine, Hershey, PA 17033, USA; echen@pennstatehealth.psu.edu (E.C.C.); rkaddis@pennstatehealth.psu.edu (R.J.K.M.); 2 Department of Microbiology & Immunology, Penn State College of Medicine, Hershey, PA 17033, USA

**Keywords:** retrovirus, RSV, RNA, dimerization, microscopy, FISH

## Abstract

Retroviruses are unique in that they package their RNA genomes as non-covalently linked dimers. Failure to dimerize their genomes results in decreased infectivity and reduced packaging of genomic RNA into virus particles. Two models of retrovirus genome dimerization have been characterized: in murine leukemia virus (MLV), genomic RNA dimerization occurs co-transcriptionally in the nucleus, resulting in the preferential formation of genome homodimers; whereas in human immunodeficiency virus (HIV-1), genomic RNA dimerization occurs in the cytoplasm and at the plasma membrane, with a random distribution of heterodimers and homodimers. Although in vitro studies have identified the genomic RNA sequences that facilitate dimerization in Rous sarcoma virus (RSV), in vivo characterization of the location and preferences of genome dimerization has not been performed. In this study, we utilized three single molecule RNA imaging approaches to visualize genome dimers of RSV in cultured quail fibroblasts. The formation of genomic RNA heterodimers within cells was dependent on the presence of the dimerization initiation site (DIS) sequence in the L3 stem. Subcellular localization analysis revealed that heterodimers were present the nucleus, cytoplasm, and at the plasma membrane, indicating that genome dimers can form in the nucleus. Furthermore, single virion analysis revealed that RSV preferentially packages genome homodimers into virus particles. Therefore, the mechanism of RSV genomic RNA dimer formation appears more similar to MLV than HIV-1.

## 1. Introduction

Retroviruses are unique in that they package their single-stranded RNA genomes as non-covalently linked dimers. Genome dimerization is evolutionarily conserved in retroviruses; mutation of the genome dimerization site or failure of a genome to appropriately dimerize results in loss of infectivity of the resulting virus [1,2,3,4,5,6]. It has been proposed that packaging of a genome dimer confers an evolutionary advantage to retroviruses (reviewed in [7,8,9,10]). During the early stage of retrovirus infection, the retroviral reverse transcriptase mediates two distinct strand transfer reactions that switch between the genomes to generate the double-stranded DNA provirus [11]. These transfer reactions facilitate recombination between the two non-identical copies of the genome, allowing for repair of damage to the fragile RNA genome or acquisition of novel mutations, both actions that encourage increased genetic diversity [7,8,9,10,11,12,13].

In vitro experiments have identified two regions of the genome that are important for retroviral genome dimerization: the dimer linkage structure (DLS) and the dimerization initiation site (DIS). The DLS was identified in Rous sarcoma virus (RSV) and characterized by electron microscopy as a physical region where the 5′ untranslated region (5′UTR) of two partially-denatured RSV genomes interacted to form a dimer structure [14,15,16,17]. Further characterization of the DLS in RSV identified a 28-nucleotide imperfect palindrome that is proposed to play a role in genomic RNA dimerization [17]. The DIS is an autocomplementary sequence located across the top of a stem-loop structure in the 5′UTR of the retrovirus genome. Two DIS structures can interact via Watson–Crick base-pairing to form a kissing-loop structure [18,19]. This initial interaction is proposed to form a “loose dimer”, which is then stabilized to form a “tight dimer” with the binding of the Gag structural protein [20,21,22,23,24]. DIS structures have been characterized in most retroviruses, as reviewed in [7].

Previous studies in cell culture have characterized two models of genomic RNA dimerization in retroviruses. In murine leukemia virus (MLV), genome dimerization occurs in the nucleus in a co-transcriptional fashion, resulting in the preferential formation of genetically identical genome homodimers [25,26,27,28,29]. In contrast, in human immunodeficiency virus type 1 (HIV-1), genomic RNA dimerization occurs in the cytoplasm and at the plasma membrane in the presence of Gag, and there is no preference for dimer composition, with homodimers and heterodimers detected with equal probability [13,30,31,32]. In both HIV-1 and MLV, the psi packaging sequence, a highly structured region of the 5′ UTR bound by the Gag protein for packaging, is located downstream of the splice donor site and is present only on unspliced viral RNA (vRNA) [33,34,35,36,37]. Additionally, in HIV-1, RNA sequences located upstream and downstream of the splice donor have been reported to contribute to selection of full-length genomic RNA by Gag for packaging into virions, and this subject remains an area of continued investigation [35,38,39,40,41]. In contrast to these two retroviruses, little is known about the subcellular location of RSV genomic RNA dimer formation or whether homo- or heterodimers are preferentially incorporated into virions. Furthermore, selective incorporation of unspliced RSV vRNA into virions [2,42] is complicated because the psi sequence is located upstream of the splice donor site and is therefore present on both spliced and unspliced vRNAs. Thus, the mechanism by which RSV Gag distinguishes between the two vRNA species remains poorly understood.

To address these questions, we used single molecule RNA labeling methods to visualize RSV unspliced vRNAs in cells and virions. We were interested in determining the subcellular localization of unspliced vRNA heterodimers that were genetically distinct and therefore distinguishable using different fluorescence markers. Notably, vRNA homodimers could not be reliably differentiated from single vRNA molecules (monomers) using these methods because they both appeared as a single-colored focus within the cell.

Using confocal microscopy, we detected RSV RNA heterodimers in the nucleus, cytoplasm, and at the plasma membrane, suggesting that genome dimerization may originate in the nucleus, similarly to MLV. Deletion of the DIS from the RSV leader sequence significantly reduced colocalization of genetically distinct unspliced vRNAs, indicating that these dual-colored foci were likely formed by the process of dimerization. Single virion analysis of genome composition demonstrated that RSV primarily packaged genomic RNA homodimers, once again similar to that of MLV. Lastly, we demonstrated that intracellular heterodimers were primarily composed of a pair of unspliced vRNAs rather than being influenced by the presence of a mixture of labeled unspliced and spliced vRNAs. Taken together, these findings define the genome dimerization preferences for RSV and suggest that dimers may initially arise in the nucleus, in an analogous fashion as MLV.

## 2. Materials and Methods

### 2.1. Plasmid Construction and Cloning

**Figure 1:**
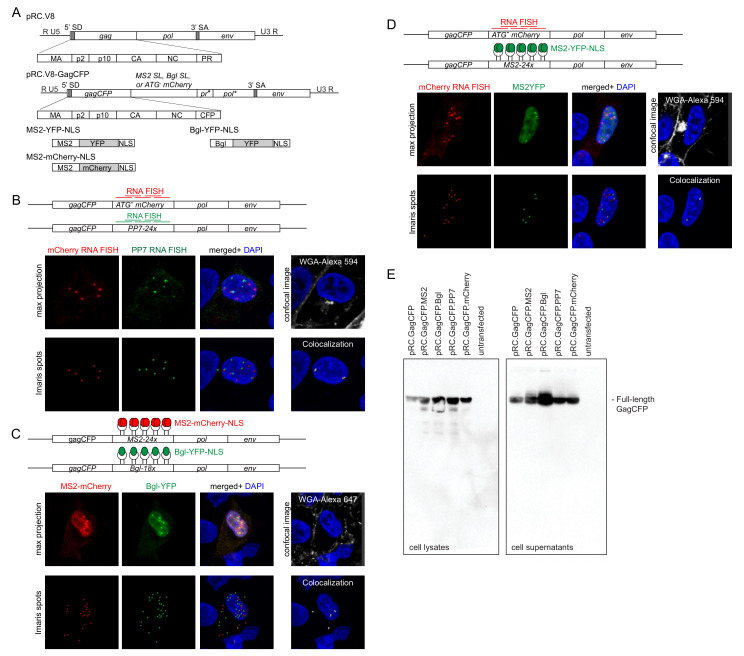
Three approaches to visualize genome dimerization in Rous Sarcoma virus (RSV). (**A**) Schematic of pRC.V8, the wild-type RSV proviral construct that forms the backbone plasmid that was modified to generate pRC.V8.GagCFP, which contains CFP fused to NC, disrupting the *gagpol* reading frame, resulting in a non-functional PR and *pol* (designated as pr* and pol*). pRC.V8.Gag-CFP contains insertion of either RNA binding stem-loops (MS2SL or BglSL) or a non-coding *mCherry* (ATG-*mCherry)* sequence. Below are schematics of the fluorophore-fused MS2 and Bgl binding proteins, which contain an NLS; (**B**) In the first approach, proviral constructs containing a noncoding *mCherry* sequence (pRC.GagCFP.ATG-mCherry) or PP7-derived sequence encoding RNA stem-loops (pRC.GagCFP.PP7) were co-transfected, each serving as a target for specific smFISH probes labeled with Alexa 647 (against *mCherry;* red) or Alexa555 (against PP7, green). WGA-Alexa 594 (gray) staining was used to outline the plasma membrane (image shown is a single z-plane); (**C**) In the second approach, proviral constructs containing the noncoding *mCherry* sequence (pRC.GagCFP.ATG-mCherry) were detected by smFISH probes labeled with Alexa 647 (red) or MS2 aptamer encoding RNA stem-loops (pRC.GagCFP.MS2) bound by the MS2-YFP protein (green). WGA-Alexa 647 staining was used to outline the plasma membrane (image shown is a single z-plane); (**D**) In the third approach, proviral constructs containing either the MS2 cassette (pRC.GagCFP.MS2) or Bgl RNA stem-loops (pRC.GagCFP.Bgl) were bound by either MS2-mCherry (red) or Bgl-YFP (green), respectively. WGA-Alexa 594 staining shown in gray outlined the plasma membrane (image is a single z-plane). In each vRNA labeling combination, only unspliced vRNAs were labeled. Representative microscopy images are shown for each condition. Images were obtained as z-stacks through the width of the cell using a 63× objective at 3.5× optical zoom. Foci were identified using the “Spot Function” in Imaris. Colocalization between RNA foci was determined as described in the Materials and Methods. Image histograms were adjusted in the Imaris software for ease of visualization; (**E**) The proviral constructs used were budding competent, as shown by immunoblotting using RSV anti-CA antibody to detect full-length Gag-CFP in cell lysates (**left**) and supernatants (**right**).

The basis for the constructs used in this paper is the replication-competent RSV proviral plasmid pRC.V8 [43], which was derived from pBH-RCAN-HiSV [44] and contains the Schmidt–Ruppin A genome with the Bryan high-titer strain *pol* gene and substitution of the pATV8 *gag* gene. The pRC.V8 plasmid only contains one splice acceptor site and generates one unspliced RNA and one spliced *env* RNA [45,46]. pRC.V8 was used as a template to generate an RSV proviral construct with a Gag-CFP fusion protein (pRC.GagCFP) using Gibson Assembly cloning (New England Biolabs, Ipswich, MA, USA). The CFP gene is located between the NC and protease (PR) sequences (Figure 1A), which disrupts the PR reading frame, eliminating cleavage of Gag-CFP. Insertion of Bgl-18x loops (pRC.GagCFP.Bgl) [30], MS2-24x loops (pRC.GagCFP.MS2) [47], PP7-24x loops (pRC.GagCFP.PP7) [48], or a non-coding ATG-*mCherry* RNA sequence (pRC.GagCFP.ATG-mCherry) isolated from N2-mCherry (Clontech, Palo Alto, CA, USA) at the end of the Gag-CFP coding region ensured that only unspliced vRNA would be labeled by smFISH probes or when the relevant fluorescently-tagged proteins were co-expressed. pCR4-24XMS2SL-stable (Addgene plasmid # 31865; http://n2t.net/addgene:31865, accessed on 10 May 2021) and pCR4-24XPP7SL (Addgene plasmid # 31864; http://n2t.net/addgene:31864, accessed on 10 May 2021) were created and deposited by Dr. Robert Singer, Albert Einstein Medical College. The pMS2-YFP-NLS construct was a kind gift from Dr. Robert Singer [49]. The pBgl-mCherry-NLS and pSL-Bgl-18x RNA stem-loop constructs were generous gifts from Dr. Wei-Shau Hu (NCI, NIH [50]). The pMS2-mCherry-NLS and pBgl-YFP-NLS constructs were cloned by exchanging the fluorophore sequences from pBgl-mCherry and pMS2-YFP, respectively. Each of the fluorophore-fused RNA binding proteins contains an NLS located after the fluorophore at the 3′ end of the construct. The DIS-deficient proviral construct (pRC.V8.∆DIS) was generated from the pGEM.RSVLTR.15-4 construct, which contains a 77-nucleotide deletion made by linearizing the plasmid with SstI followed by digestion with nuclease BAL-31 [2]. The DIS-deleted sequence was cloned into pRC.V8 using Gibson assembly [51], as previously described [52]. The pRC.V8.∆DIS and pRC.V8 constructs were modified to contain either MS2-24x RNA stem-loops or a noncoding *mCherry* RNA sequence inserted into the *ClaI* (New England Biolabs, Ipswich, MA, USA) site after *env*. At this location, the MS2-24x loops and non-coding *mCherry* RNA sequence would label both unspliced and spliced vRNA.

### 2.2. Cell Culture, Transfection, Slide Preparation, and Virus Particle Purification

Experiments were performed in QT6 cells, a chemically transformed quail fibroblast cell line [53] maintained in F10 medium supplemented with 10% fetal calf serum, chicken serum, penicillin/streptomycin, and Fungizone and cultured at 38.5 °C in 5% CO_2_. For microscopy, cells were grown directly on 1.5 mm glass coverslips placed in 35 mm cell culture dishes. Transient transfections of the proviral constructs in QT6 cells were performed using the calcium phosphate method, using 3 µg of each proviral DNA construct and 0.5 µg of each respective fluorophore-fused RNA binding protein, when applicable. Fresh media was added 16–18 h post-transfection to allow for recovery and coverslips were harvested 24 h post-transfection. To visualize plasma membranes, WGA staining was performed by adding 1 mL of 5.0 µg/mL fluorophore-conjugated WGA (ThermoFisherScientific, Waltham, MA, USA) diluted in 1X PBS for 10 min at 37 °C, followed by washing coverslips twice with 1X PBS, per manufacturer’s instructions. WGA-Alexa 647 was used in cells co-expressing with MS2-mCherry and Bgl-YFP. WGA-Alexa 594 was used in cells treated with mCherry and PP7 smFISH probes labeled with Alexa 647 and Alexa 555, respectively, or with MS2-YFP. These fluorophore combinations were chosen to eliminate crosstalk during imaging. Coverslips were then fixed in 3.7% paraformaldehyde in 2X PHEM buffer (120 mM PIPES, 55 mM HEPES, 20 mM EGTA, and 16.5 mM MgSO4, pH to 7.0) [54] for 15 min at room temperature and washed in 1X PBS prior to DAPI staining to visualize nuclei. Fixed and stained coverslips were mounted on glass slides using ProLong Diamond Antifade Mountant (ThermoFisherScientific, Waltham, MA, USA) and cured at room temperature in the dark for 72 h before imaging. Coverslips for smFISH were treated as described below in the section titled “Probe preparation and single molecule fluorescence in situ hybridization (smFISH)”.

For single virion analysis, QT6 cells grown in 60 mm cell culture dishes were transfected with 4 µg of each proviral construct and 0.75 µg of the respective fluorophore-fused RNA binding protein. After 16–18 h, cells were washed in 1X PBS and fresh media added. Supernatants were harvested 24 h later. Cell debris was removed from the supernatants by centrifugation at 2000 rpm for 10 min at 4 °C. The medium was then clarified through a 0.45 µm syringe filter to remove cellular debris. Virus particles were concentrated by centrifuging through a 20% *w*/*v* sucrose cushion at 13,000 rpm for 2.5 h at 4 °C. The virus particles were resuspended in 20 µL PBS and spotted onto a gelatin-coated coverslip [32]. Coverslips were fixed in 3.7% formaldehyde/PBS for 15 min at room temperature, washed with PBS, DAPI stained, mounted onto glass slides in antifade reagent (ThermoFisher Scientific, Waltham, MA, USA) and sealed with clear nail polish.

### 2.3. Immunoblot Analysis

Cell lysates and virions were harvested 24 h post-transfection. Transfected cells were collected and lysates in RIPA buffer (50 mM Tris-HCl (pH 7.2), 150 mM NaCl, 1% Triton X-100, 0.01% deoxycholate, and 0.1% SDS). The lysed cells were centrifuged at 16,000× *g* for 30 min to clear the lysates. Virions were harvested from cell supernatant as described above and resuspended in 10 ul 4x loading dye. Twenty-five micrograms of cell lysates (determined by Bradford assay) and the viral pellets were separated by SDS-PAGE and transferred to polyvinylidene difluoride (PVDF) membrane. Immunoblotting was performed using mouse anti-RSV capsid antibody (a gift of Dr. Neil Christensen, Penn State College of Medicine) and detected by chemiluminescence.

### 2.4. Probe Preparation and Single Molecule Fluorescence in Situ Hybridization (smFISH)

RNA smFISH probes complementary to the PP7 RNA stem-loops were created by linearizing the pCR4-24XPP7SL plasmid with BamHI (New England Biolabs, Ipswich, MA, USA) and performing in vitro transcription with T7 RNA polymerase (ThermoFisher Scientific, Waltham, MA, USA) and incorporating amino-allyl labeled dUTPs (ThermoFisher Scientific, Waltham, MA, USA) to generate single-stranded RNA. The probes were then labeled with Alexa555 dye (ThermoFisher Scientific, Waltham, MA, USA) according to manufacturer’s instruction, purified, and concentrated (Zymo Research, Orange, CA, USA). RNA smFISH probes against the non-coding *mCherry* sequence were designed and purchased from Stellaris (LGC Biosearch Technologies, Novato, CA, USA).

smFISH was performed according to Stellaris protocol [55]. Briefly, cells on coverslips were fixed in 3.7% formaldehyde/PBS for 15 min, washed in PBS. To visualize the plasma membrane, coverslips were stained with wheat germ agglutinin WGA-Alexa594 (ThermoFisher Scientific-Invitrogen, Waltham, MA, USA) for 12 min at room temperature and washed twice with PBS. The coverslips were then permeabilized overnight in 70% ethanol at 4 °C. The next morning, cells were rehydrated in wash buffer (2X SSPE, 10% formamide). smFISH probes were diluted in hybridization buffer (2X SSPE, 10% formamide, 10% dextran sulfate) and coverslips incubated overnight in a humid chamber at 37°C. The next morning, cells were washed and DAPI stained (1:1000, diluted in wash buffer) before mounting on glass slides and cured with ProLong Diamond Antifade Mountant (ThermoFisher Scientific-Invitrogen, Waltham, MA, USA) for 72 h.

### 2.5. Microscopy, Image Acquisition, Image Analysis

Microscope images were obtained on a Leica SP8 confocal microscope (Leica Microsystems, Buffalo Grove, IL, USA) with a 63X oil-objective, using UV, white light, or argon lasers. Z-stacks were obtained at a thickness of 0.3 µm, through the width of the nucleus (as indicated by DAPI staining) or through the width of the cell (as indicated by WGA staining). Single virion imaging was performed at 6X optical zoom with a pinhole size of 2.5 AU.

To analyze images, the location of vRNA foci in z-stack images was determined by using the Imaris Spots function (Bitplane, Concord, MA, USA). The Spots were placed in the center of each vRNA focus as determined by pixel intensity and manually determined thresholds. Different imaging methods resulted in foci of different intensities, therefore image histograms were adjusted as needed to visualize fainter foci. Colocalization between Imaris Spots was determined using the “Find colocalized spots” function in Imaris. Colocalization was determined in three dimensions using a threshold value to measure the extent of overlap between the previously determined Spots in the center of the vRNA foci: a threshold of 0.5 µm detected an overlap of no more than 0.5 µm between the centers of adjacent Spots. The colocalization threshold value of 0.5 µm was selected to accommodate the resolution limits of the 63x objective on the Leica SP8 confocal microscope in three dimensions: the x,y resolution limit is 0.25 µm and the z resolution limit is 0.75 µm. The imaging and analyses were performed in a blinded manner. For single virion analysis, a colocalization threshold of 0.25 µm was first used to determine colocalization between the two genomes because the images were obtained in the x,y plane, which has a resolution limit of 0.25 µm. To determine whether an identified dimer also colocalized with Gag-CFP, the colocalization threshold was set to a maximum distance of 0.5 µm to accommodate the size of the genome dimer focus.

To analyze the subcellular location of colocalized foci, the Imaris Cell function was used to partition the nucleus from the cytoplasm based upon DAPI staining and place Spots in the nuclear and non-nuclear cellular compartments independently. This analysis allowed for independent adjustment of image histograms in each subcellular compartment to optimally visualize fainter foci. A 3D Surface of the plasma membrane was then created using the WGA staining as a mask. Colocalized genome foci were considered to be located at the plasma membrane if they were within 0.25 µm of the WGA Surface measured in any dimension.

## 3. Results

### 3.1. Three Imaging Combinations to Visualize Genome Heterodimers within Cells

To detect genome heterodimers composed of two genetically distinct unspliced vRNAs within cells, we used three complementary approaches using a combination of two techniques: one to selectively label vRNA using smFISH and the second using fluorescently-labeled proteins bound to aptamer sequences inserted into vRNA. We created genetically distinguishable genomes that could be identified by being labeled by distinct fluorophores, and those differentially labeled RNAs that colocalized were considered to be heterodimers. By contrast, homodimers or monomeric RNA molecules were indistinguishable using this imaging method, as either moiety would appear as a single-colored RNA focus. Our methodology was based on prior imaging studies of genomic RNA dimers performed in other retroviruses [30,32,33]. For our experiments, we used a combination of smFISH and RNA stem-loop aptamer systems (MS2, Bgl, and PP7) to distinctly label differentially-tagged RSV unspliced RNAs, and previous studies demonstrated that these labeling techniques allow for visualization of RNA in cells at single molecule resolution [30,55,56,57].

Schematic diagrams of the provirus plasmids and fluorophore-tagged coat proteins used in our work are shown in Figure 1. For each provirus plasmid, either RNA binding stem-loops or a non-coding *mCherry* RNA sequence was inserted after the *gag* gene in the RSV proviral construct, allowing for labeling of only the unspliced vRNA. None of these proviral constructs was infectious due to the fusion of CFP to the C-terminus of the Gag protein, located between NC and PR; as a result, the PR was non-functional and there was no maturation of Gag.

In the first experiment, we used two distinct sets of smFISH probes complementary to either the inserted noncoding *mCherry* sequence, in which the initiator AUG codon was deleted (pRC.GagCFP.ATG-mCherry), or a cassette encoding 24-copies of the PP7 RNA binding stem-loops (pRC.GagCFP.PP7) (Figure 1A) [48,58]. In the second approach, we used a set of smFISH probes targeting the noncoding *mCherry* RNA sequence in pRC.GagCFP.ATG-mCherry and co-expressed pRC.GagCFP.MS2, which has 24 tandem repeats of the MS2 RNA stem-loops [47], with a plasmid encoding MS2-YFP-NLS to label the unspliced vRNA (Figure 1B). The third set of experiments used two distinct RNA-loop binding aptamers: 24 copies of the MS2 RNA stem-loops in pRC.GagCFP.MS2 or 18 copies of the Bgl RNA sequence in pRC.GagCFP.Bgl, which were co-expressed with pMS2-mCherry-NLS and pBgl-YFP-NLS (Figure 1C) [30,47] to visualize two distinct RSV unspliced vRNA populations.

To visualize unspliced vRNA foci throughout the cell, a z-series was obtained using confocal microscopy through the width of the cell as defined by WGA staining of plasma membranes. Foci representing unspliced vRNA fluorescent signals were identified using the Imaris software Spots function, which incorporates pixel intensity and a manually defined threshold to label foci in three dimensions (Figure 1). It is important to note that each focus of unspliced vRNA labeled by a single color could be either (a) unspliced vRNA used as a translating mRNA in the cytoplasm, (b) a newly transcribed monomer of unspliced vRNA in the nucleus, or (c) a genomic RNA homodimer, and each of these populations is indistinguishable using our imaging methods. By contrast, dual-colored, colocalized unspliced vRNA foci were categorized as genomic RNA heterodimers. To analyze the images using an unbiased approach, colocalized foci were identified using the Imaris colocalization program in three dimensions between the centers of the unspliced vRNA foci, as described in Materials and Methods. Insertion of the RNA-loops or non-coding *mCherry* sequence does not interfere with virus assembly, as each of the proviral constructs used was budding competent and can generate virus particles detected by immunoblotting of cell lysates and supernatants (Figure 1E).

The total number of colocalized dual-colored (red and green) unspliced RNA heterodimers and single-color RNA foci (red or green) per cell were measured using each of the three imaging combinations (Table 1). The total number of unspliced vRNA foci labeled within each cell was comparable among the three imaging approaches used (1474 foci for approach 1; 1662 foci for approach 2; and 1727 foci for approach 3). Of note, the smFISH-smFISH technique detected fewer colocalized RNA foci compared to the other techniques, although each unspliced vRNA population was labeled equally, with no apparent difference between the amounts of non-colocalized red or green foci. In comparison, the MS2-mCherry and Bgl-YFP stem-loop systems labeled unspliced vRNA molecules unequally, with a bias toward more efficient Bgl-YFP labeling, resulting in more vRNA foci visualized. The imaging approaches also varied slightly in terms of total percentages of unspliced vRNA heterodimers visualized, calculated as the total number of red-green colocalized foci detected divided by the total number of vRNA foci per imaging combination (6.2, 9.2, and 16.4%, respectively) (Table 1).

### 3.2. Visualization of RSV Genome Heterodimers in Different Subcellular Compartments

**Figure 2:**
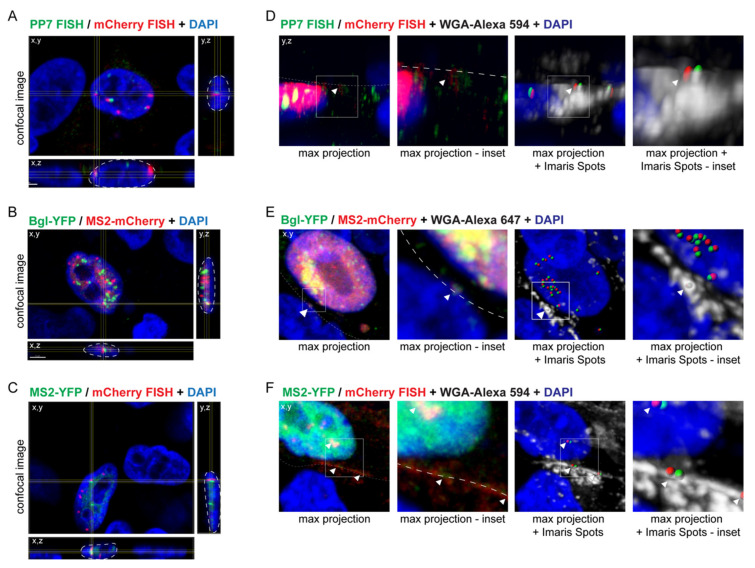
Examples of genome heterodimers located in the nucleus and at the plasma membrane. Shown are representative images of genome heterodimers within the nucleus (**A**–**C**), as defined by DAPI staining (blue), and at the plasma membrane, as defined by WGA-Alexa 594 staining (**D**,**E**) or Alexa 647 staining (**F**) in gray, for each of the described imaging methods. See Materials and Methods for further detail on the selection of the appropriate WGA conjugate. For the images showing genome heterodimers within the nucleus, the crosshairs expand into x,z and y,z planes to show the genome heterodimer is located within the DAPI staining in three dimensions (as outlined in white dashed line). For the images showing genome heterodimers at the plasma membrane, the two images on the left are a max projection image and inset showing the genome heterodimer at the plasma membrane (outline in white dashed line), while the two images on the right show the WGA-Alexa 594 or WGA-Alexa 647 staining (in gray) with Imaris Spots representing genome heterodimers. White arrowheads mark representative genome heterodimers at the plasma membrane (**D**,**E**) and at the plasma membrane and in the nucleus (**F**). Image histograms were adjusted in the Imaris software for ease of visualization for the purpose of this manuscript. Images were obtained using a 63× objective at a 3.5× optical zoom.

To examine where genomic RNA dimerization occurs within the cell, the average number of genomic RNA heterodimers present in the nucleus, in the cytoplasm, and at the plasma membrane was quantitated (Table 1). Colocalization with DAPI staining was used as a marker for genome heterodimers in the nucleus, and WGA staining was used as a marker for genome heterodimers located at the plasma membrane (Figure 2). Either WGA-Alexa 594 or WGA-Alexa 647 was used to visualize the plasma membrane, depending on the other fluorophores expressed, see Materials and Methods for more details. Using DAPI and WGA staining as a mask, we were able to determine the number of genome heterodimers located in the nucleus and at the plasma membrane, respectively. Representative images of genome heterodimers located within the nucleus and at the plasma membrane are shown in Figure 2. The number of genome heterodimers in the cytoplasm were calculated as the number of genome heterodimers that were located neither within the nucleus nor at the plasma membrane. All three imaging combinations identified genome heterodimers within the nucleus, representing 67.4, 50.3, and 47.0% of total number of cellular genome heterodimers, respectively, suggesting that RSV genomic RNA dimers form in the nucleus, possibly by a similar mechanism as previously defined for MLV [25,26,27,28]. It is not known whether the heterodimers formed in the nucleus were subsequently transported to the cytoplasm and plasma membrane, or alternatively, whether heterodimers seen in the cytoplasm and plasma membrane formed at these locations. On average, more genomic RNA heterodimers were visualized in the nucleus than in the cytoplasm or at the plasma membrane when using smFISH-smFISH or MS2-smFISH combinations, whereas approximately equal numbers of genomic RNA heterodimers were seen between the nucleus and cytoplasm when using the MS2 and Bgl aptamer RNA labeling techniques. It should be noted that genome foci visualized with smFISH were brighter in the nucleus, likely due to accumulation of multiple copies of genomic vRNA at transcription sites, and fainter in the cytoplasm and at the plasma membrane, where lower numbers of genomic vRNA were likely present. These differences in intensity of the vRNA foci may have affected quantitative Imaris Spot analysis, possibly resulting in uneven detection of vRNA foci throughout the subcellular compartments, since an intensity threshold had to be defined prior to the Spot function analysis.

### 3.3. Genomic RNA Heterodimer Formation Depends on the Presence of the DIS

**Figure 3:**
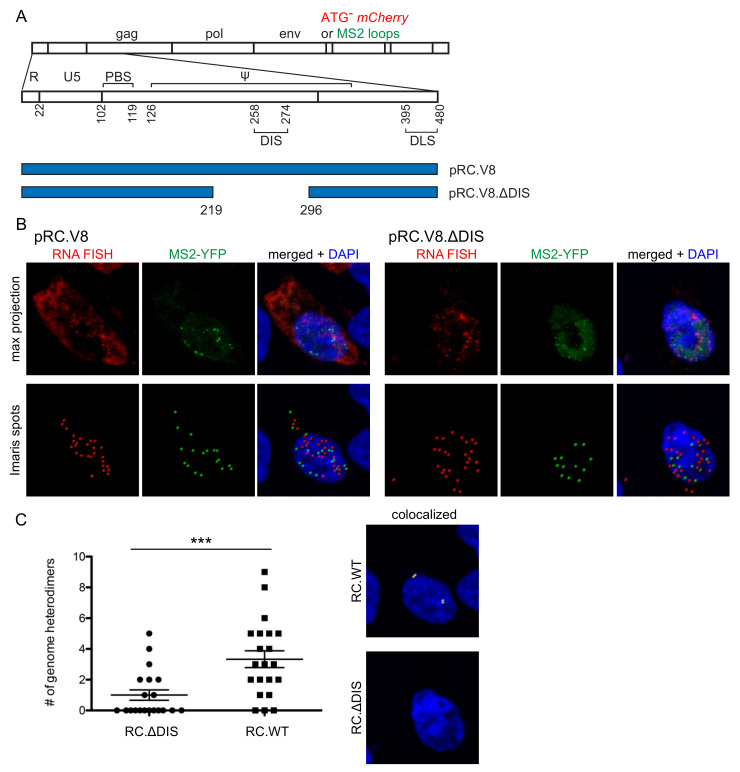
Genome heterodimer formation dependent on presence of dimerization initiation signal (DIS) (**A**) Schematic of pRC.V8 and pRC.V8.∆DIS proviral constructs, which contains a 77-nucleotide deletion (bp 219-296) removing the L3 stem-loop DIS. Each construct contains either 24x-MS2 RNA stem-loops or a non-coding *mCherry* RNA sequence after *env*; (**B**) Cells on the left were co-transfected with pRC.V8.ATG-mCherry, pRC.V8.MS2, and MS2-YFP (green) and subjected to *mCherry* RNA smFISH (red). On the right, cells were transfected with pRC.∆DIS.ATG-mCherry, pRC.∆DIS.MS2, and MS2-YFP (green) and *mCherry* RNA smFISH (red) was performed. Cells were imaged through the width of the nucleus, as assessed by DAPI staining, using a 63× objective at 3.5× optical zoom. The top rows of each panel show maximum projection images and the bottom row images indicate the location of foci identified using the Imaris Spots function; (**C**) Colocalization of dual-colored foci was analyzed using the Imaris Spots colocalization function (colocalization threshold 0.5 µm). Statistical significance was assessed by using negative binomial regression model, and a total of 30 cells were analyzed in two biological replicates, (*** *p* < 0.0001). Histograms were adjusted in the Imaris software for optimal visualization.

In RSV, base-pairing between two DIS sequences on interacting genomes allows for the formation of a genomic RNA dimer [18,59]. Our laboratory and others previously demonstrated that deletion of the DIS sequence results in reduced genomic RNA dimers in vitro [2,17,60]. Therefore, we sought to examine the importance of the DIS in the formation of genomic RNA heterodimers in vivo using our genomic RNA visualization methods. We compared a full-length proviral construct (pRC.V8) with a proviral construct containing a deleted DIS (pRC.V8.ΔDIS); in each construct, a noncoding ATG-*mCherry* RNA sequence or a cassette of 24 copies of MS2 stem-loops was inserted into the intron to label unspliced vRNA (Figure 3A). Using a combination of *mCherry* RNA smFISH and MS2 coat protein RNA labeling methods, we used confocal microscopy to determine whether heterodimer formation in cells was dependent on the presence of the DIS (Figure 3).

Our findings revealed that deletion of the DIS resulted in the formation of significantly fewer genome heterodimers as compared to wild-type (n = 30 cells, *p* < 0.0001) (Figure 3C). Two important conclusions can be drawn from these results. First, this in vivo result aligns with previous in vitro studies demonstrating that this region of the RSV leader sequence is important for dimer formation [2,14,16,17,18,59,60,61,62]. Second, this finding validates the specificity of our study to quantitate genomic RNA heterodimers within the cell using aptamer binding loops and RNA smFISH imaging techniques. We can conclude that the colocalization of RNA foci visualized in the imaging experiments was most likely due to intermolecular interactions between the distinct RNA genomes and not as a result of transient or non-specific interactions between the fluorescent tags, randomly overlapping Spots, or other imaging artifacts.

### 3.4. Genome Dimer Preferences in RSV

**Figure 4:**
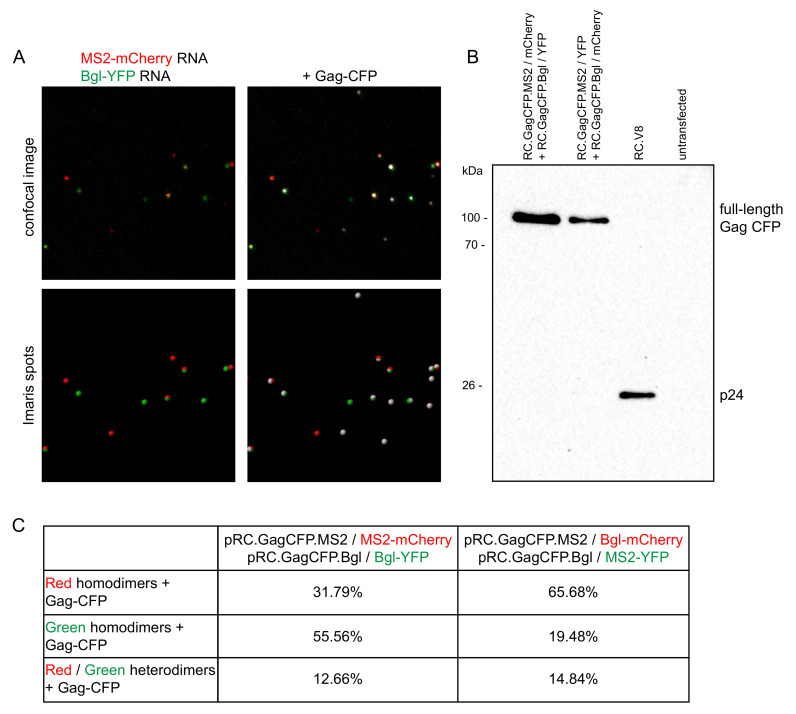
Single virion analysis in RSV shows preferential formation of genome homodimers: (**A**) Confocal microscopy of virions harvested from cell supernatant. QT6 fibroblasts were transiently transfected with proviruses containing MS2 and Bgl RNA stem-loop-containing and their respective fluorophore-labeled coat proteins. Visualized RNA genome foci are considered to be genome dimers, as they were present in virions harvested from the supernatant of transiently transfected cells. The top row shows the confocal microscopy images and the bottom row shows Spots labeled using the Imaris software. Images of virions were obtained using a 63× objective at 6× optical zoom; (**B**) Detection of Gag-CFP from virions harvested from cell supernatant expressing loop-containing provirus and their coat proteins by immunoblotting. The Gag-CFP is uncleaved, as expected, since CFP is located between NC and protease. pRC.V8, the wild-type parental proviral construct, which has normal protease activity, was used as a positive control for virus budding; (**C**) Quantitation of the number of genome homodimers and genome heterodimers present within harvested virions. Only RNA genomes that colocalized with Gag-CFP were analyzed, to ensure that RNA was located within a virion. A red-only or green-only focus was considered to be a genome homodimer and a red-green colocalized focus was considered to be a genome heterodimer. The colocalization threshold was set to 0.25 µm to determine red-green colocalization. To determine colocalization of an RNA focus with a Gag-CFP in virus particles, a colocalization threshold of 0.5 µm was used. Image histograms were adjusted in the Imaris software to optimize visualization.

To determine whether RSV has a packaging preference for genome homo- or heterodimers, single virion imaging analysis was performed [30,32]. Equivalent amounts of proviral plasmid DNA (pRC.GagCFP.MS2 and pRC.GagCFP.Bgl) and their respective fluorophore-fused coat proteins were co-expressed in QT6 cells. The genomes were identical except for the sequence of the RNA stem-loop cassettes inserted. Twenty-four hours post-transfection, virions were harvested from the supernatant and concentrated through a sucrose gradient. The proviral constructs contain Gag-CFP, so we used the CFP channel to detect virus particles (Figure 4A). Only red and green foci that colocalized with a Gag-CFP focus were considered for analysis to ensure that only genome dimers within virus particles were counted. Assuming that each virion contains two copies of genomic RNA, red-green colocalized foci were considered to be genome heterodimers and single-color foci were considered to be genome homodimers [30,32]. Virus budding was confirmed by immunoblotting (Figure 4B). In the proviral constructs, the CFP was fused to Gag between the NC domain and protease, resulting in loss of protease activity and lack of maturation of Gag products. Previous studies have already verified that minimal indiscriminate binding occurs between the MS2 and Bgl RNA labeling systems [30,31].

Assuming random formation of genomic RNA dimers based on the Hardy–Weinberg equation, we would have expected to observe 50% homodimers and 50% heterodimers [25,26,27,30,32]. However, in 1035 virions analyzed (Figure 4C), we visualized 12.66% genome heterodimers and 87.35% genome homodimers (MS2-mCherry and Bgl-YFP), suggesting preferential formation of genome homodimers. To verify our results and consider the possibility of differences in the affinities of binding of MS2 versus Bgl to their respective stem-loops, we exchanged the fluorophores on the coat proteins (MS2-YFP and Bgl-mCherry). Even with swapped fluorophores, genome homodimers were the predominant population (14.84% genome heterodimers and 85.16% genome homodimers) among 539 virus particles analyzed. Although we observed similar heterodimer and homodimer percentages between the two conditions, it should be noted that we again observed a labeling bias in that genomes containing Bgl-18x stem-loops (RC.GagCFP.Bgl) that colocalized with Gag-CFP were seen in higher numbers than genomes containing MS2-24x (RC.GagCFP.MS2).

### 3.5. Spliced vRNA Does Not Contribute to Intracellular Heterodimers in RSV

**Figure 5:**
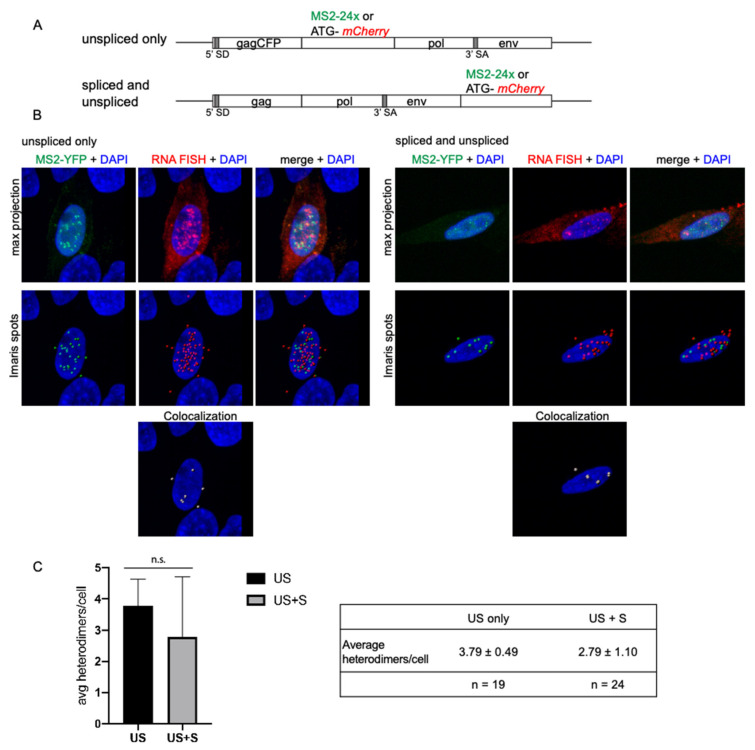
Contribution of spliced vRNA to intracellular genome heterodimers in RSV: (**A**) Schematic of the provirus constructs used to visualize only unspliced vRNAs (top) or spliced and unspliced vRNAs (bottom). Each proviral construct contains either a MS2-24x loop cassette or a non-coding *mCherry* RNA sequence used for RNA smFISH. The location of the 5′ splice donor (5′ SD) and 3′ splice acceptor (3′ SA) are indicated; (**B**) Representative confocal microscopy images of genome heterodimers in cells co-transfected with the proviral constructs containing *ATG-mCherry* (red) and MS2-24x loop cassette and MS2-YFP (green) are shown here. Cells were imaged by a z-stack through the width of the cell, as determined by WGA membrane staining, using a 63× objective at 3.5× optical zoom. A colocalization threshold of 0.5 µm was used to determine whether two Spots were colocalized; (**C**) Comparison of the average number of heterodimers per cell between only unspliced vRNAs and spliced and unspliced vRNAs (*n* = 19 cells and *n* = 22 cells, respectively). The average number of heterodimers per cell is 3.79 ± 0.49 in unspliced only and 2.79 ± 1.10 in spliced and unspliced vRNAs. Significant outliers were identified and removed using the Grubbs’ test (*p* > 0.05, alpha = 0.5). No significant difference in the average number of genome heterodimers per cell was detected by unpaired t-test (*p* = 0.2132). Image histograms were adjusted in the Imaris software for optimal visualization.

Previous studies demonstrated that small amounts of spliced vRNA are packaged into RSV virions at a ratio of 195.3:1 (unspliced vRNA to *env* mRNA) [42]. Spliced RSV vRNA makes up a smaller population within virus particles, even though it contains the same packaging and dimerization signals in the leader sequence as the unspliced vRNA [63], so we were interested in determining whether we would observe a change in the amount of heterodimers detected in cells when both unspliced and spliced RNA could be visualized in cells. By changing the location of the MS2-24x stem-loop cassette or noncoding ATG-*mCherry* sequence to the *env* spliced subgenomic mRNA sequence, we selectively imaged only unspliced vRNAs versus both unspliced and spliced vRNAs (Figure 5A). Confocal z-stack images were obtained that spanned the entire cell, as determined by WGA staining (data not shown) to detect the plasma membrane (Figure 5B).

On a single cell basis, the average number of heterodimers per cell was 3.79 ± 0.49 for unspliced vRNA and 2.79 ± 1.10 for spliced plus unspliced vRNAs (*n* = 19 cells and 24 cells, respectively; *p* = 0.2132; Figure 5C). These data demonstrated that there was no significant difference in genome heterodimer formation when only unspliced vRNA was detected compared to labeling both spliced and unspliced vRNAs, suggesting that spliced vRNAs do not contribute to vRNA heterodimer formation within cells to a significant degree.

## 4. Discussion

Previous studies have characterized two in vivo modes of genome dimerization: in MLV, genome dimerization occurs in the nucleus in a co-transcriptional fashion that results in preferential formation of genome homodimers [25,26,27,28,29], whereas in HIV-1, genome dimerization occurs in the cytoplasm and at the plasma membrane, and genome dimers are generated randomly [13,30,31,32,33]. However, there had previously been no studies that characterized the in vivo mechanism of genome dimerization in RSV, which we examined in this study. Previous studies from our laboratory demonstrated that trafficking of RSV Gag through the nucleus is required for efficient genomic RNA packaging, and ribonucleoprotein complexes composed of Gag and unspliced vRNA form in the nucleus [2,4,64,65,66,67,68,69,70]. In RSV, the DIS sequence is present on both unspliced and spliced vRNAs, further complicating the mechanism governing preferential packaging of unspliced RSV genomic RNA dimers into virions. Therefore, we tested the hypothesis that RSV genome dimerization occurs in the nucleus, possibly in a co-transcriptional manner in a fashion analogous to MLV.

To examine this idea, we visualized unspliced vRNA heterodimers in three subcellular compartments (Figure 1 and Figure 2). The presence of RSV genome heterodimers in the nucleus indicated that dimerization can occur within the nucleus. The heterodimers visualized in the cytoplasm and at the plasma membrane may have initially formed in the nucleus and then trafficked to the cytoplasm and plasma membrane, or they could be newly formed in each of these subcellular compartments. Our imaging technique cannot distinguish between the two possibilities, and live cell imaging with particle tracking would be required to address this interesting question. Our data also suggest that genome dimerization in RSV may be similar to the mechanism used by MLV, in which unspliced vRNAs arising in the nucleus undergo dimerization co-transcriptionally [25,26,27,28]. This conclusion is supported by the single virion analysis demonstrating that RSV preferentially packages genomic RNA homodimers. One finding that is inconsistent with RSV forming homodimers more frequently than heterodimers is the observation that RSV has a high recombination rate, which would implicate two genetically different vRNAs forming packaged dimers [71]. Further studies will be required to reconcile these differences and to determine whether RSV genome dimerization occurs in a co-transcriptional manner, similar to MLV. Of note, in these imaging experiments we are not able to distinguish between genome homodimers and monomers within cells. This is the reason we chose to analyze only the genome heterodimer population in cells with respect to subcellular localization. It is important to note that vRNA visualized in our study were detected using methods that detect single RNA species, although each vRNA focus may contain more than one vRNA molecule. Therefore, brighter foci may contain more copies of vRNA, irrespective of whether smFISH or aptamer binding methods were used.

For HIV, it has been reported that genome dimerization occurs at the plasma membrane in the presence of Gag [31,32,33]. Ferrer et al. demonstrated that the percentage of HIV genome heterodimers visualized increased fourfold, moving from the cytoplasm to the plasma membrane and into virions (from 10 to ~40%) [32]. The increase in heterodimers was proposed to be due to the presence of Gag at the plasma membrane, facilitating genome dimerization and packaging into newly formed virus particles. By contrast, in our study of RSV, the percentages of genome heterodimers visualized using the MS2 and Bgl labeling systems were approximately equal in the nucleus and in the cytoplasm (approximately 47% each, Table 1) with only 5% of genomic RNA heterodimers located at the plasma membrane. One possibility is that dimers formed in the nucleus and cytoplasm could remain loosely associated as “immature dimers” [60,72]. Once genomic RNA dimers traffic to the plasma membrane, they may be stabilized as they are incorporated into assembling virus particles and processing of the Gag precursor occurs [73,74,75]. Another possibility is that genome incorporation and budding from the plasma membrane occurs more rapidly in RSV compared to HIV, resulting in our seeing fewer RSV heterodimers at the plasma membrane under steady-state conditions. Live cell imaging studies will be needed to visualize the kinetics of RSV genomic RNA dimer formation within each subcellular compartment and subsequent trafficking of the viral ribonucleoprotein complex to the site of virus particle release at the plasma membrane.

This report is the first in vivo visualization of the role of the DIS sequence in genome dimerization using a full-length RSV genome. The sequences required for dimerization in the RSV leader sequence were previously identified using in vitro dimerization assays with truncated vRNAs that contained the 5′ UTR and minimal sequences in the *gag* coding region [18,60,61,62]. Importantly, in our study, deletion of the DIS sequence (Figure 3) significantly reduced the number of genome heterodimers visualized, indicating that wild-type RSV heterodimers were likely forming through specific intermolecular interactions (Figure 3) rather than due to transient, non-specific interactions between the two vRNA genomes or the inserted aptamer sequences. Additionally, this result also addresses the concern that colocalization does not necessarily correlate to an intermolecular interaction. It should be noted that the DIS deletion we tested did not completely abolish genome dimerization, as a low level of genome dimers was still visualized, both in our imaging analysis and in previously published in vitro dimerization assays [2,18]. This finding suggests that additional regions of the genome may play a role in genome dimerization, possibly in initiation of genome dimer formation or by stabilizing interactions to strengthen the dimer linkage. In the experiments described here, the DLS sequence in the *gag* coding region was left intact to ensure expression of a functional Gag protein, and therefore the DLS could contribute to the low levels of genome dimerization. These findings warrant further study to define the minimal sequence required for genomic RNA dimerization in the context of the otherwise full-length genome. A recent study that utilized SHAPE to characterize secondary structures in the RSV 5′-leader RNA identified a novel secondary site that may play a role in genomic RNA dimerization and packaging [52]. It is unlikely that the inserted sequences (RNA binding loops or non-coding *mCherry* sequence) contributed to genome dimerization, as the sequences have been used in previous studies for vRNA labeling and dimerization [30,47,48,76].

As mentioned previously, the RSV psi packaging sequence is located upstream of the 5′ splice acceptor site and is thus present on both spliced and unspliced vRNAs. RSV unspliced vRNA is preferentially packaged into newly formed virions, and studies have suggested that the genome dimerization is linked to genome packaging [2,5,8,36]. It is thought that the HIV-1 5′UTR forms a conformation that facilitates Gag binding to psi while promoting dimerization of the genomes [77]. There is a possibility that the RSV 5′UTR adopts a similar conformation to promote a link between packaging and dimerization. Furthermore, data from our laboratory suggest that RSV Gag nuclear trafficking is linked to efficient genomic RNA packaging, and we have visualized interaction of Gag with genomic RNA in the nucleus [70]; however, it is currently unclear whether Gag binding to psi precedes dimerization or if Gag preferentially binds to psi in the context of an RNA dimer. Further work needs to be done to answer this question.

We applied our imaging techniques to characterize the genome composition of genome heterodimers within cells when either only unspliced vRNAs or both spliced and unspliced vRNAs were labeled with different fluorescent markers (Figure 5). We found that there was no difference in the mean number of genome heterodimers formed when two unspliced vRNA genomes or both spliced and unspliced vRNAs were expressed within cells. The presence of spliced vRNA genomes did not significantly affect vRNA heterodimer frequency, suggesting that heterodimers are composed primarily of unspliced vRNAs. Nikolaitchik et al. demonstrated that HIV-1 genome dimers were packaged based on recognition of the dimer structure [37], and perhaps a similar mechanism occurs in RSV.

Our studies also demonstrated that each imaging technique has unique characteristics and caveats. As previously noted, the smFISH technique resulted in detection of brighter RNA foci in the nucleus, likely due to accumulation of multiple copies of genomic vRNAs, and fainter RNA foci in the cytoplasm and at the plasma membrane, likely due to the presence of fewer copies of genomic vRNAs. The difference in intensity resulted in uneven detection by the Imaris Spots algorithm, which uses an intensity threshold to identify foci. This is likely the explanation for the decreased number of total colocalized red-green foci detected by smFISH-smFISH as compared to MS2-YFP-smFISH and MS2-YFP-Bgl-YFP (92, 153, and 283, respectively). The use of MS2 and Bgl aptamer labeling systems resulted in higher levels of nonspecific background fluorescence due to the presence of fluorophore-tagged coat proteins that were not bound to vRNA. We also noticed a labeling bias between the MS2 and Bgl aptamers, with more Bgl-labeled foci visualized as compared to MS2-labeled foci intracellularly and within virions. The bias was not due to the nature of the fluorophore fused to the coat protein because when the fluorophores were swapped, a Bgl labeling bias was still seen (Table 1). All of these issues were avoided by using smFISH to label both genomes, and similar overall results were obtained in all of our experiments, suggesting that the above caveats associated with the MS2, PP7, and Bgl labeling systems did not significantly influence our findings.

In summary, the results of this study demonstrate that RSV genomic RNA dimer formation occurs in the nucleus, and dimers may also arise in the cytoplasm and at the plasma membrane. Genome heterodimer formation is dependent on the presence of the L3 stem-loop and palindromic DIS sequence and appear to be composed of a pair of unspliced vRNAs. Additionally, we demonstrated the use of three distinct imaging approaches to visualize distinct single molecule RNA genomes in cells. The presence of genome dimers in the nucleus is intriguing considering that RSV ribonucleoprotein complexes arise in the nucleus and nuclear Gag is important for efficient packaging of genomic RNA dimers [2,4,64,65,67]. A recent study demonstrated that HIV Gag is also present in the nucleus in complex with unspliced vRNA [78]. These findings warrant additional experiments to determine whether the nuclear population of RSV Gag facilitates genomic RNA dimerization, and these studies may serve as a framework for investigating the role of Gag nuclear trafficking in the replication of other retroviruses.

## Figures and Tables

**Table 1:** Quantitation analysis of subcellular localization of genome heterodimers using three imaging combinations. The total number of non-colocalized red genomic RNA foci, non-colocalized green genomic RNA foci, and colocalized red/green genomic RNA foci per cell are shown. The percent of total heterodimers was calculated assuming that singly-colored unspliced vRNA foci present in the cell were genomic RNA homodimers. Genomic RNA foci were identified using the Imaris Spots function with a manually defined intensity threshold. Genomic RNA heterodimers were then quantified using the Imaris “Find colocalized spots” function with a threshold of 0.5 µm, as described in Materials and Methods. To assess the subcellular location of genomic RNA heterodimers within subcellular compartments, DAPI staining was used as a mask to distinguish the confines of the nucleus and WGA staining was used as a mask to identify the plasma membrane.

	*mCherry* smFISH + *PP7* smFISH	*mCherry* smFISH + MS2-YFP	MS2-mCherry + Bgl-YFP
Total red non-colocalized foci	590	1063	504
Total green non-colocalized foci	792	446	940
Total red/green colocalized foci	92	153	283
Total number of foci analyzed	1474	1662	1727
Total % red non-colocalized foci	40.0%	64.0%	29.2%
Total % green non-colocalized foci	53.7%	26.8%	54.4%
Total % red/green colocalized foci	6.2%	9.2%	16.4%
Total % colocalized in nucleus	67.4%	50.3%	47.0%
Total % colocalized in cytoplasm	28.3%	45.8%	51.2%
Total % colocalized at plasma membrane	4.3%	3.9%	1.8%
# cells	24	27	24

## Data Availability

Data are present within the article.
